# Supplementary material for: TEtrimmer: a tool to automate the manual curation of transposable elements
Source: Nat Commun. 2025 Sep 25;16:8429. doi: 10.1038/s41467-025-63889-y (PMC12462492; doi:10.1038/s41467-025-63889-y)
Supplement: Supplementary file 1 — Supplementary Information [file 41467_2025_63889_MOESM1_ESM.pdf]

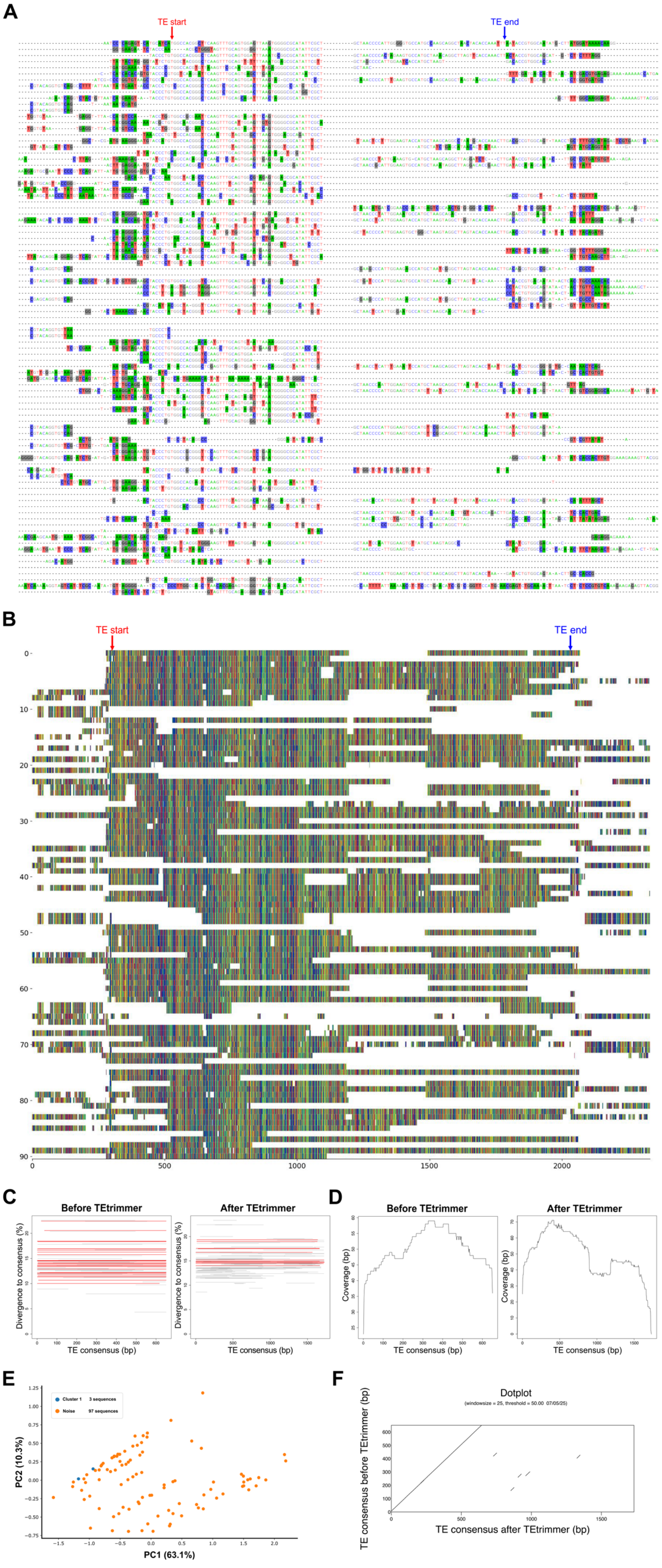
A
TE start
TE end
B
TE start
TE end
0
10
20
30
40
50
60
70
80
90
0
500
1000
1500
2000
C
Before TEtrimmer
After TEtrimmer
Divergence to consensus (%)
TE consensus (bp)
Divergence to consensus (%)
TE consensus (bp)
D
Before TEtrimmer
After TEtrimmer
Coverage (bp)
TE consensus (bp)
Coverage (bp)
TE consensus (bp)
E
Cluster 1 3 sequences
Noise 97 sequences
PC2 (10.3%)
PC1 (63.1%)
F
Dotplot
(windowsize = 25, threshold = 50.00 07/05/25)
TE consensus before TEtrimmer (bp)
TE consensus after TEtrimmer (bp)

Supplementary Figure 1. TEtrimmer can correctly cluster MSAs derived from divergent and fragmented TEs. The *B. hordei* DNA transposon named rnd_1_family_420, initially annotated by RepeatModeler2 and re-analysed with TEtrimmer, is shown as an example for a relatively old and fragmented TE. **A** The alignment of 91 sequences in total shows the TE boundary regions of the MSA (100 nucleotide sites are displayed for each side) after TEtrimmer analysis. Nucleotide background colors in the MSA represent sites where the proportion of the respective nucleotide is below 0.4. The TE boundaries defined by TEtrimmer are indicated as Start crop point (red) and End crop point (blue). The two boundary regions were artificially connected by ten gaps (-), and the total length of the MSA is indicated on the top. **B** The plot shows the entire MSA (91 sequences) after TEtrimmer analysis. The TE boundaries are indicated as in (A). Nucleotides are represented with colored bars (nucleotide A, green; C, blue; G, black; T, red); gaps are indicated as blank regions in the plot. The *x*-axis gives the nucleotide position (in bp) within the MSA. **C** and **D** The panels show TE-Aid plots before and after TEtrimmer analysis. **C** The panels show a BLASTN plot before (left panel) and after (right panel) TEtrimmer analysis. The *x*-axis indicates the TE consensus nucleotide position (in bp), and the *y*-axis is the sequence divergence in percent compared to the TE consensus sequence. Each line indicates a BLASTN hit; red lines highlight hits with a sequence divergence below 1.5% and a sequence coverage >90%. **D** Self-dot plots before (left panel) and after (right panel) TEtrimmer analysis. The axes show the nucleotide position (in bp) in the TE consensus sequence. **E** The PCA plot was calculated based on the respective phylogenetic tree relative branch distance matrix. The members of the two clusters are indicated by blue (cluster 1) and orange (noise cluster) dots, and the number of sequences within each cluster is indicated. The axes represent the principal components PC1 and PC2. **F** Dot plots (window size = 25 bp, threshold = 50; the threshold represents the minimal sum of substitution scores in a defined window required for a dot to be plotted) of the TE consensus sequence before (*y*-axis) and after (*x*-axis) TEtrimmer analysis. Axes show the nucleotide position (in bp). Identical regions are represented by short diagonal lines outside the main diagonal.

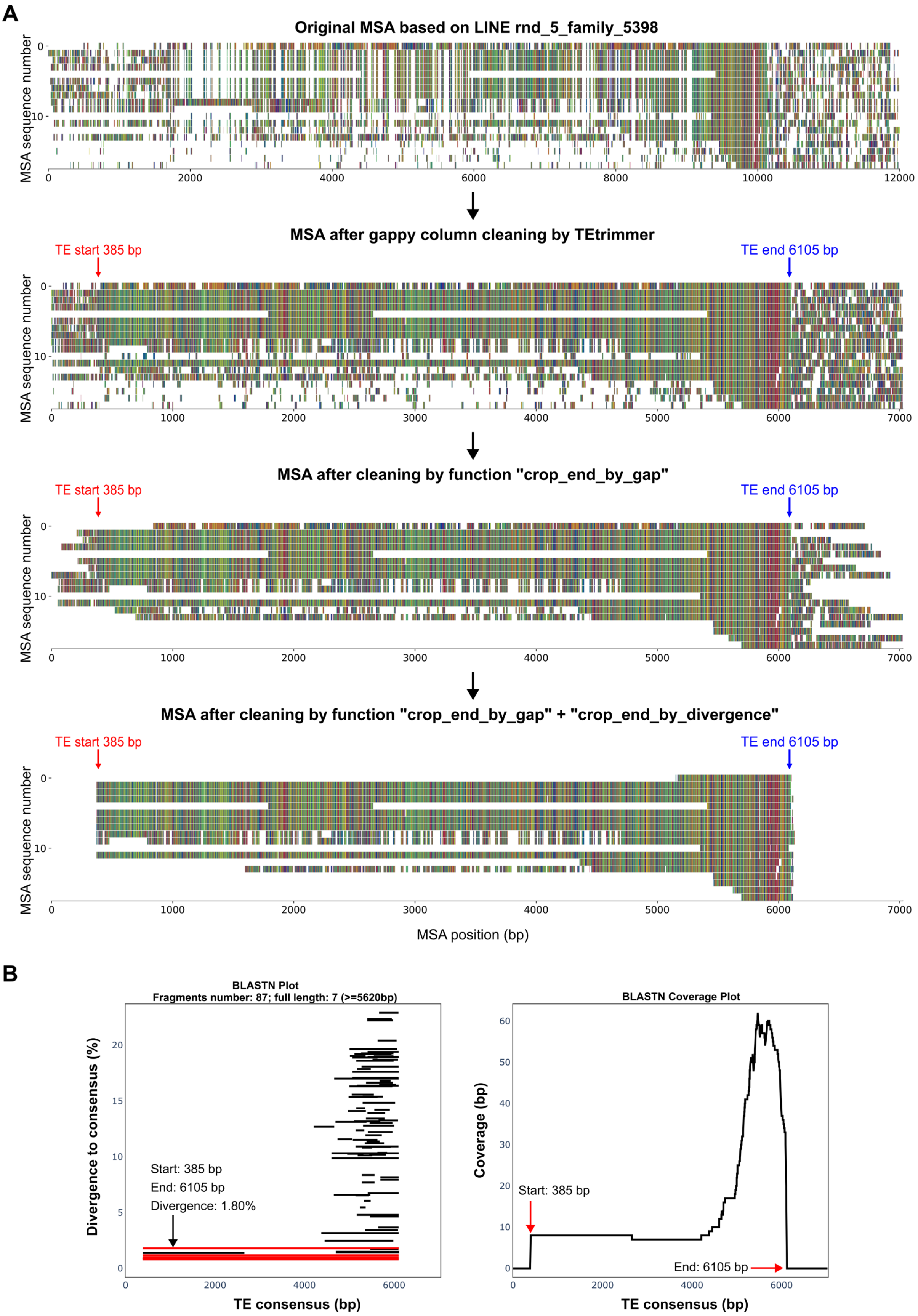

Supplementary Figure 2. TEtrimmer can accurately clean lowly conserved regions from MSAs of more divergent LINE elements with fewer intact copies. A *D. rerio* LINE element (named rnd_5_family_5398), identified by RepeatModeler2, was chosen as another example for

demonstrating the performance of TEtrimmer MSA cleaning. **A** Following a BLASTN search of the selected LINE sequence against the *D. rerio* genome, sequence extension, and MSA generation, the original MSA before cleaning is shown in the top panel. Nucleotides are represented with colored bars (nucleotide A, green; C, blue; G, black; T, red); gaps are indicated as blank regions in the plots. The original MSA before cleaning contains many gappy columns and noisy rows. After MSA column cleaning by the TEtrimmer function `remove_gap_columns`, the majority of the gappy columns are removed (second panel). Then, TEtrimmer cleans sequences in the MSA row by row using the TEtrimmer functions `crop_end_by_gap` and `crop_end_by_divergence` (bottom two panels), which removes lowly conserved regions (bottom panel). The LINE boundary positions were indicated by TE start 385 bp (red) and TE end 6105 bp (blue). **B** TE consensus sequences were generated based on the cleaned MSA from A (bottom panel), and a BLASTN search was conducted for this consensus sequence against the *D. rerio* genome. The left panel shows a BLASTN match result plot. The *x*-axis indicates the TE consensus nucleotide position (in bp), and the *y*-axis is the sequence divergence in percent compared to the TE consensus sequence. Each line indicates a BLASTN hit; red lines highlight hits with a sequence coverage >80%. The full-length BLASTN hit *x*-axis coordinates are indicated with the black arrow. The right panel represents the BLASTN coverage plot. The *x*-axis indicates the TE consensus nucleotide position (in bp), and the *y*-axis is the coverage depth (in bp). The coverage values of each nucleotide in the TE consensus sequence are shown as a black line.

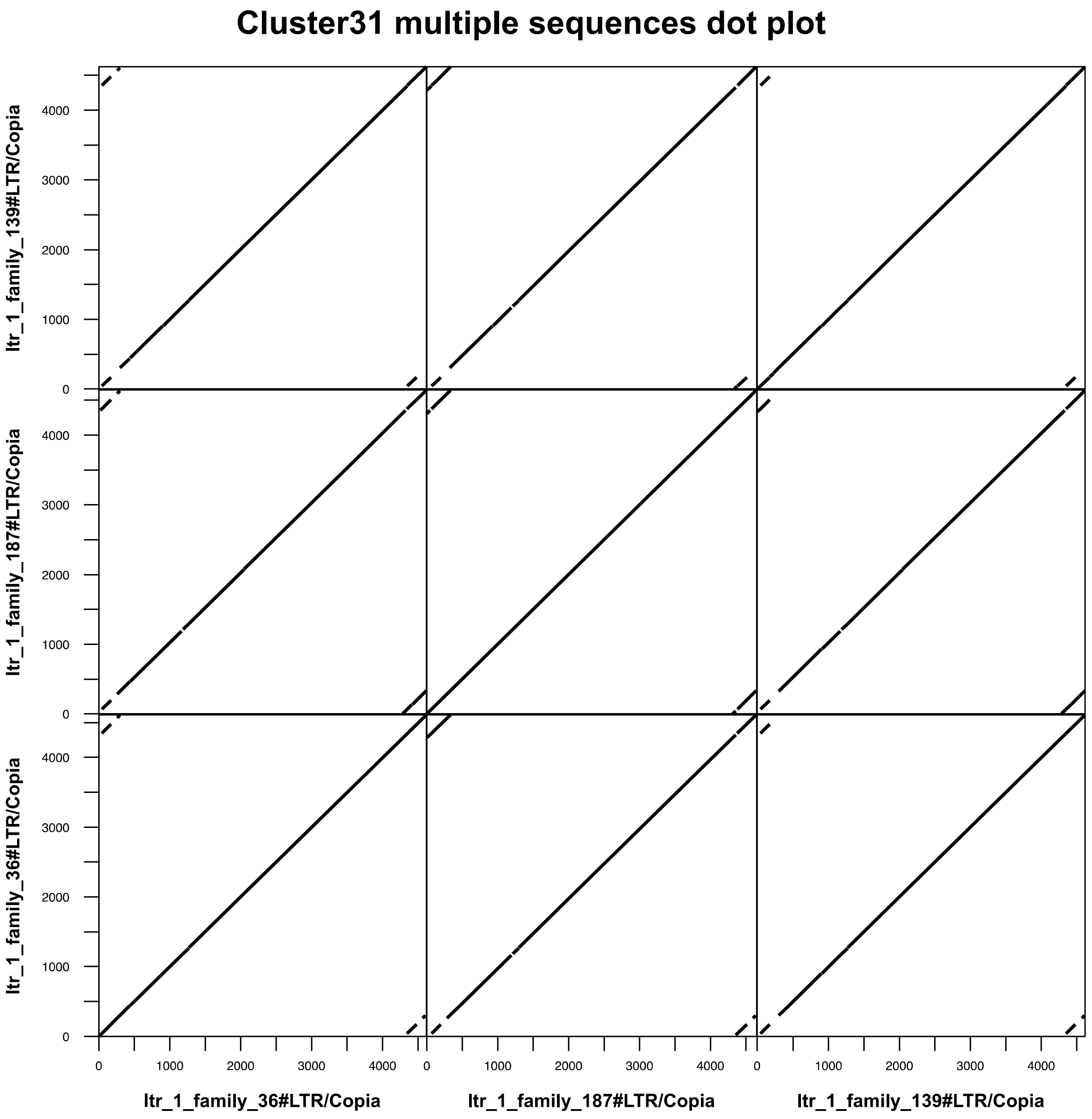

Supplementary Figure 3. Multiple sequence dot plot based on all sequences in a TE cluster. The multiple sequence dot plot (window size = 25 bp, threshold = 50; the threshold represents the minimal sum of substitution scores in a defined window required for a dot to be plotted) shows identical regions among sequences. Axes show the nucleotide position (in bp) of each sequence. Identical regions are represented by diagonal lines.

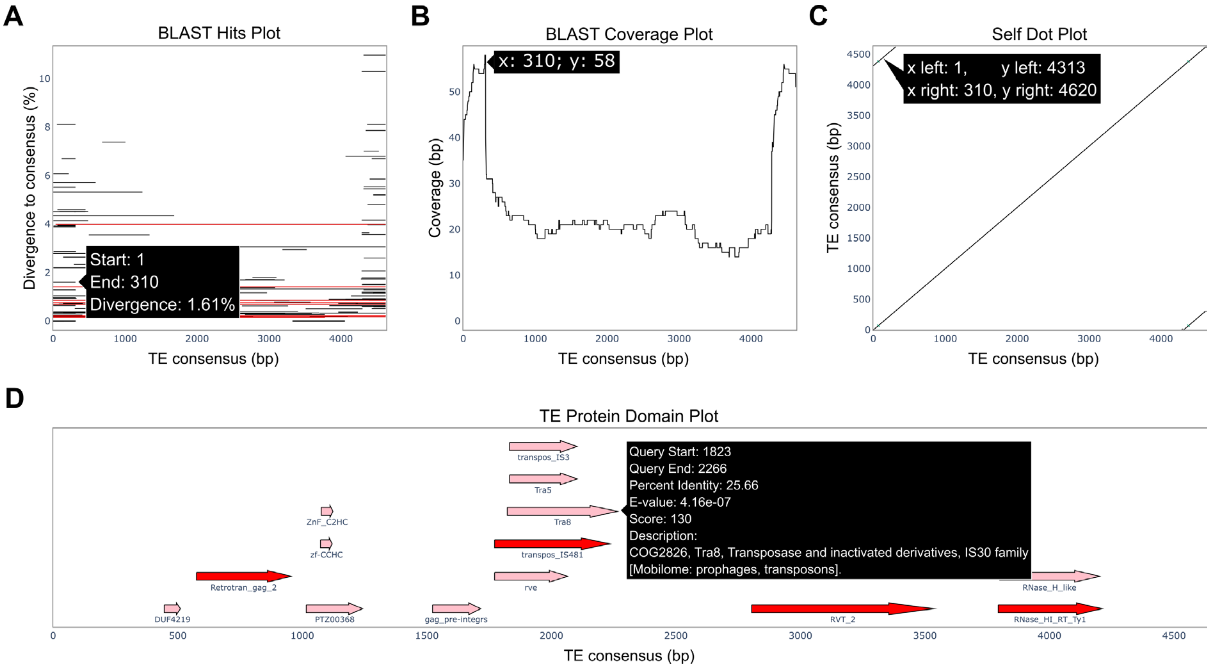

Supplementary Figure 4. The TEtrimmer GUI application generates interactive reporting plots for TE consensus sequences. A The BLASTN hit plot. BLASTN search is performed for the TE consensus sequence against the corresponding genome. Each line indicates a BLASTN hit; red lines highlight hits with a sequence divergence below 1.5% and a sequence coverage >90% compared to the TE consensus sequence. The *x*-axis indicates the TE consensus nucleotide position (in bp), and the *y*-axis is the sequence divergence in percent. Hovering over a BLASTN hit reveals its specific location along the TE consensus sequence. **B** The BLASTN coverage plot visualizes the hit density across each nucleotide position of the TE consensus sequence. The *x*-axis represents the TE sequence, and the *y*-axis indicates the coverage depth (in bp). Precise coverage information for each position can be retrieved by hovering over the corresponding region. **C** Self-dot plots for the TE sequence. Both axes show the nucleotide position (in bp) along the TE consensus sequence. Repetitive regions appear as short diagonal lines outside the main diagonal. Hovering over these regions reveals their precise coordinates. **D** The TEtrimmer GUI integrates the conserved domain database (CDD) to detect protein domains within the TE consensus sequence. The direction and position of each identified protein domain are represented by colored arrows. The *x*-axis shows the nucleotide position (in bp) along the TE consensus sequence. Detailed descriptions of each protein domain can be retrieved by hovering over the corresponding arrow.

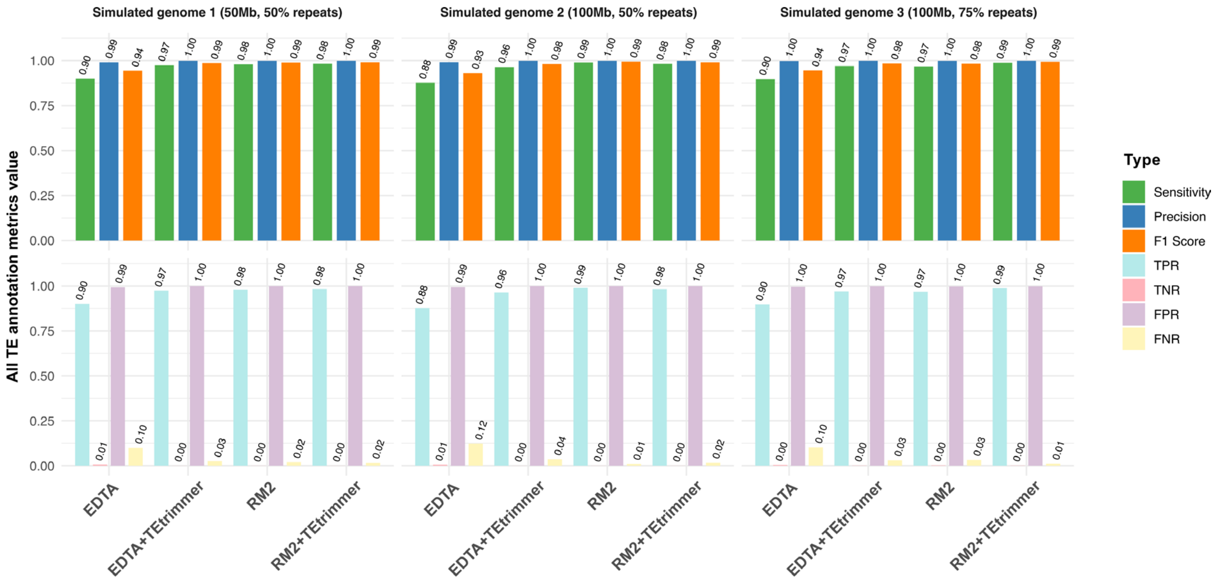

Supplementary Figure 5. Genome-wide TE annotation performance of EDTA2, RepeatModeler2 (RM2), and both after additional TEtrimmer analysis (EDTA2+TEtrimmer and RM2+TEtrimmer, respectively) based on simulated genomes. Simulated genomes were created as described by Rodriguez & Makałowski, targeting 50 Mb and 50% repeats, 100 Mb and 50% repeats, and 100 Mb and 75% repeats, respectively. All genome-wide TE annotation results based on *de novo*-generated libraries were compared with the original genome annotation file created during the simulation of genomes. Upper panel: Sensitivity (green), precision (blue), and F1 score (orange) were calculated with a confusion matrix for the three indicated simulated genomes. Lower panel: True positive rate (TPR, pastel mint), true negative rate (TNR, pastel coral), false positive rate (FPR, pastel lavender), and false negative rate (FNR, butter-cream) were calculated as described by Rodriguez & Makałowski (2022). Each bar represents an individually obtained value (N=1).

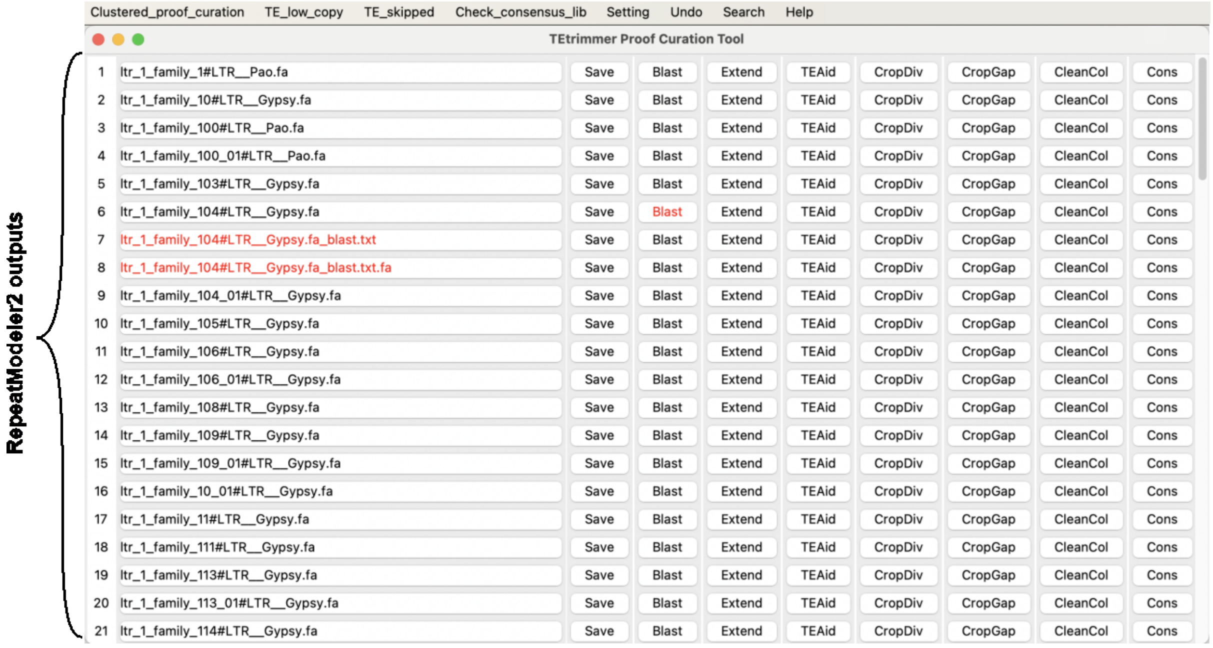

Supplementary Figure 6. The TEtrimmer GUI application helps to inspect and improve any TE consensus libraries. All TE consensus sequences from RepeatModeler2 are listed in the left region of the GUI. Users can inspect each file by selecting the corresponding file name. BLASTN search of consensus sequences based on relative genomes can be performed by activating the Blast button. This function extracts the corresponding BLAST hit sequences and stores them in a file, which is shown in the left panel of the GUI. The extracted sequences can be aligned for MSA generation. TE sequences in the MSA can be extended by activating the Extend button. The TEAid button helps to generate the interactive report plot (see Figure 4 and Supplementary Figure 3). The buttons CropDiv, CropGap, and CleanCol relate to the MSA cleaning functions `crop_end_by_divergence`, `crop_end_by_gap`, and `remove_gap_columns`, respectively. The button Cons can be used to generate a consensus sequence based on the corresponding MSA.

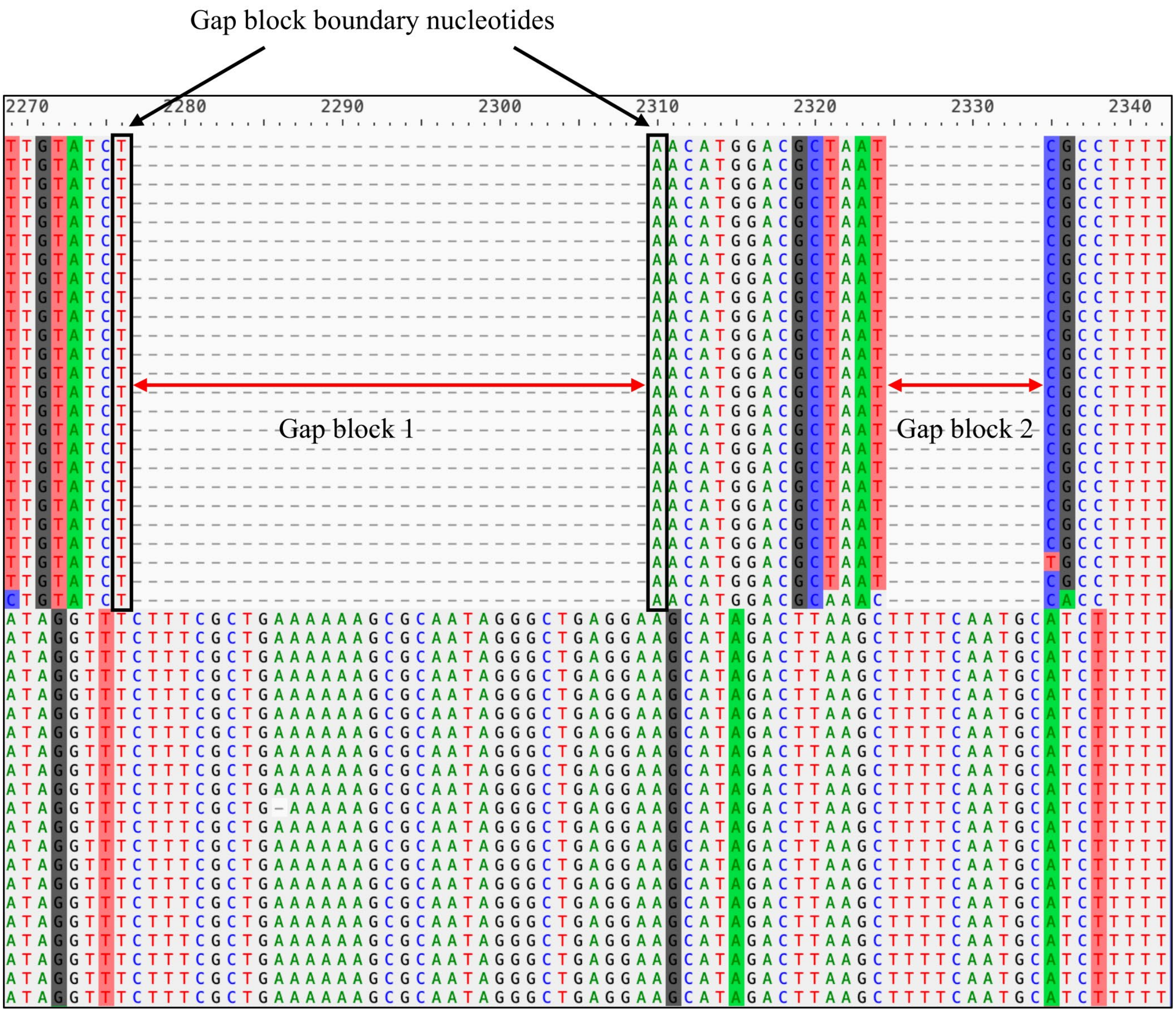

Supplementary Figure 7. TEtrimmer utilizes gap information from MSA to assist in clustering TE sequences. Multiple sequence alignment of TE fragments highlighting indel-driven gap blocks. Nucleotides and nucleotide backgrounds are colored as follows: A, green, C, blue, G, black, T, red; the nucleotide background colors in the MSA represent sites where the proportion of the respective nucleotide is below 0.4. A contiguous gap block of indel-rich columns is denoted by the red arrow, and its conserved flanking boundaries are marked by the black arrow and square.

Supplementary Tables.

Supplementary Table 1. Criteria for evaluation levels of the TEtrimmer outputs.

Category	Presence of terminal repeats	Classified	Sequence number in the MSA	Number of full-length BLASTN hits	Presence of PFAM domain
Perfect	TRUE	TRUE	≥30	≥5	TRUE
Good	TRUE	Not required	≥10	≥2	Not required
Reco_check	Not required	Not required	≥20	≥2	Not required
Need_check	Not required	Not required	≥10	Not required	Not required
Low_copy	TRUE	Not required	< 10	≥2	Not required

These criteria might not efficiently evaluate TEs lacking terminal repeats such as LINEs and SINEs.

Supplementary Table 2. Confusion matrix calculation for the MSA cleaning performance.

Category	True positive site	False negative site	False positive site	True negative site
Original MSA	Nucleotide	Nucleotide	Nucleotide	Nucleotide
Manually cleaned MSA	Gap	Gap	Nucleotide	Nucleotide
TEtrimmer-cleaned MSA	Gap	Nucleotide	Gap	Nucleotide

Supplementary Table 3. An exemplary report table generated by TEtrimmer.

Input name	Consensus name	Blast hits	Cons[1] MSA seq	Cons[1] full BLAST n	Input length	Cons[1] length	Input TE type	Reclassified TE type	Terminal repeat	Low copy	Evaluation	Status
ltr_1_family_22	ltr_1_family_22	122	89	77	9508	4947	LTR/Copia	LTR/Copia	LTR	FALSE	Perfect	processed
rnd_1_family_174	rnd_1_family_174	181	19	13	3554	5635	Unknown	LTR/Gypsy	LTR	FALSE	Good	processed
rnd_1_family_174	rnd_1_family_174_01	181	18	7	3554	5661	Unknown	LTR/Gypsy	LTR	FALSE	Good	processed
rnd_1_family_386	rnd_1_family_386	305	29	8	3769	5871	LINE/Tad1	LINE/Tad1	FALSE	FALSE	Reco_check	processed
rnd_1_family_386	rnd_1_family_386_01	305	29	6	3769	6044	LINE/Tad1	LINE/Tad1	FALSE	FALSE	Reco_check	processed
rnd_1_family_317	rnd_1_family_317	108	76	0	358	714	Unknown	Unknown	FALSE	FALSE	Need_check	processed
rnd_4_family_345	rnd_4_family_345	15	NaN	NaN	1935	1935	Unknown	Unknown	FALSE	FALSE	NaN	skipped

[1]Cons, consensus
