# Supplementary material for: TEtrimmer: a tool to automate the manual curation of transposable elements
Source: Nat Commun. 2025 Sep 25;16:8429. doi: 10.1038/s41467-025-63889-y (PMC12462492; doi:10.1038/s41467-025-63889-y)
Supplement: Supplementary file 3 — Reporting Summary [file 41467_2025_63889_MOESM3_ESM.pdf]

nature portfolio

Corresponding author(s): Ralph Panstruga, Stefan Kusch

Last updated by author(s): Aug 1, 2025

Reporting Summary

Nature Portfolio wishes to improve the reproducibility of the work that we publish. This form provides structure for consistency and transparency in reporting. For further information on Nature Portfolio policies, see our Editorial Policies and the Editorial Policy Checklist.

Statistics

For all statistical analyses, confirm that the following items are present in the figure legend, table legend, main text, or Methods section.

n/a	Confirmed	
☐	☒	The exact sample size (*n*) for each experimental group/condition, given as a discrete number and unit of measurement
☒	☐	A statement on whether measurements were taken from distinct samples or whether the same sample was measured repeatedly
☒	☐	The statistical test(s) used AND whether they are one- or two-sided<br>*Only common tests should be described solely by name; describe more complex techniques in the Methods section.*
☒	☐	A description of all covariates tested
☒	☐	A description of any assumptions or corrections, such as tests of normality and adjustment for multiple comparisons
☐	☒	A full description of the statistical parameters including central tendency (e.g. means) or other basic estimates (e.g. regression coefficient) AND variation (e.g. standard deviation) or associated estimates of uncertainty (e.g. confidence intervals)
☒	☐	For null hypothesis testing, the test statistic (e.g. *F*, *t*, *r*) with confidence intervals, effect sizes, degrees of freedom and *P* value noted<br>*Give P values as exact values whenever suitable.*
☒	☐	For Bayesian analysis, information on the choice of priors and Markov chain Monte Carlo settings
☒	☐	For hierarchical and complex designs, identification of the appropriate level for tests and full reporting of outcomes
☒	☐	Estimates of effect sizes (e.g. Cohen's *d*, Pearson's *r*), indicating how they were calculated

Our web collection on statistics for biologists contains articles on many of the points above.

Software and code

Policy information about availability of computer code

Data collection	The code of TEtrimmer v1.5.1 can be downloaded from GitHub at https://github.com/qjiangzhao/TEtrimmer and is also available via Zenodo (https://doi.org/10.5281/zenodo.16682752).
Data analysis	The code of TEtrimmer v1.5.1 can be downloaded from GitHub at https://github.com/qjiangzhao/TEtrimmer and is also available via Zenodo (https://doi.org/10.5281/zenodo.16682752).

For manuscripts utilizing custom algorithms or software that are central to the research but not yet described in published literature, software must be made available to editors and reviewers. We strongly encourage code deposition in a community repository (e.g. GitHub). See the Nature Portfolio guidelines for submitting code & software for further information.

Data

Policy information about availability of data

All manuscripts must include a data availability statement. This statement should provide the following information, where applicable:

- Accession codes, unique identifiers, or web links for publicly available datasets
- A description of any restrictions on data availability
- For clinical datasets or third party data, please ensure that the statement adheres to our policy

Data availability:

TEtrimmer is an open-source software under the license GPLv3. We used large language models, ChatGPT and GPT4, to facilitate the development of TEtrimmer. A detailed TEtrimmer manual is supplied . All benchmarking results and scripts can be found on GitHub at https://github.com/qjiangzhao/TEtrimmerPaperFile. All third-party software included in the TEtrimmer package are published under open-source licenses, with the exception of TE-Aid, for which we have approval by the author Clément Goubert (University of Arizona, USA). Source data are provided with this paper and can be found in the Source data file.

Code availability:

The TEtrimmer code (v1.5.1) can be downloaded from GitHub at https://github.com/qjiangzhao/TEtrimmer or from Zenodo at https://doi.org/10.5281/zenodo.16682752.

Research involving human participants, their data, or biological material

Policy information about studies with human participants or human data. See also policy information about sex, gender (identity/presentation), and sexual orientation and race, ethnicity and racism.

Reporting on sex and gender	Not applicable.
Reporting on race, ethnicity, or other socially relevant groupings	Not applicable.
Population characteristics	Not applicable.
Recruitment	Not applicable.
Ethics oversight	Not applicable.

Note that full information on the approval of the study protocol must also be provided in the manuscript.

Field-specific reporting

Please select the one below that is the best fit for your research. If you are not sure, read the appropriate sections before making your selection.

[x] Life sciences [ ] Behavioural & social sciences [ ] Ecological, evolutionary & environmental sciences

For a reference copy of the document with all sections, see nature.com/documents/nr-reporting-summary-flat.pdf

Life sciences study design

All studies must disclose on these points even when the disclosure is negative.

Sample size	Our study reports the development of a new bioinformatic tool. Thus, most Figures relate to the exemplary demonstration of features of this tool. "Sample size" is not an issue in our study in most cases as we did not perform experiments in a classical sense. In Figure 3C, however, we used 10 example TE sequences to create the line plots. We consider this sample size sufficient as the 10 example sequences yielded very similar outcomes (Figures 3C). No statistical method was used to predetermine sample size.
Data exclusions	No data were excluded from the analyses.
Replication	Our study reports the development of a new bioinformatic tool. Thus, "replication" is not an issue for most Figures, except where TEtrimmer was tested on eukaryotic and simulated genomes. In the case of the simulated genomes, three independent "replicates" (= simulated genomes) were analyzed. We consider this replication sufficient as the three simulated genomes yielded very similar outcomes (Figures 6 and 7). No statistical method was used to predetermine replication.
Randomization	Our study reports the development of a new bioinformatic tool. Thus, most Figures relate to the exemplary demonstration of features of this tool. "Randomization" is not an issue in our study as we did not perform experiments in a classical sense. The experiments were not randomized.
Blinding	Our study reports the development of a new bioinformatic tool. Thus, most Figures relate to the exemplary demonstration of features of this tool. "Blinding" is not an issue in our study as we did not perform experiments in a classical sense. The Investigators were not blinded to allocation during experiments and outcome assessment.

Reporting for specific materials, systems and methods

We require information from authors about some types of materials, experimental systems and methods used in many studies. Here, indicate whether each material, system or method listed is relevant to your study. If you are not sure if a list item applies to your research, read the appropriate section before selecting a response.

Materials & experimental systems

n/a	Involved in the study
☒	☐ Antibodies
☒	☐ Eukaryotic cell lines
☒	☐ Palaeontology and archaeology
☒	☐ Animals and other organisms
☒	☐ Clinical data
☒	☐ Dual use research of concern
☒	☐ Plants

Methods

n/a	Involved in the study
☒	☐ ChIP-seq
☒	☐ Flow cytometry
☒	☐ MRI-based neuroimaging

Plants

Seed stocks	Not applicable.
Novel plant genotypes	Not applicable.
Authentication	Not applicable.
